# A flood-based information flow analysis and network minimization method for gene regulatory networks

**DOI:** 10.1186/1471-2105-14-137

**Published:** 2013-04-24

**Authors:** Andreas Pavlogiannis, Vadim Mozhayskiy, Ilias Tagkopoulos

**Affiliations:** 1 Department of Computer Science, University of California Davis, One Shields Avenue, Davis, CA 95616, USA; 2 UC Davis Genome Center, University of California Davis, One Shields Avenue, Davis, CA 95616, USA

**Keywords:** Network flood, Network flux, Information flow, Gene regulatory networks, Network minimization

## Abstract

**Background:**

Biological networks tend to have high interconnectivity, complex topologies and multiple types of interactions. This renders difficult the identification of sub-networks that are involved in condition- specific responses. In addition, we generally lack scalable methods that can reveal the information flow in gene regulatory and biochemical pathways. Doing so will help us to identify key participants and paths under specific environmental and cellular context.

**Results:**

This paper introduces the theory of *network flooding*, which aims to address the problem of network minimization and regulatory information flow in gene regulatory networks. Given a regulatory biological network, a set of source (input) nodes and optionally a set of sink (output) nodes, our task is to find (a) the minimal sub-network that encodes the regulatory program involving all input and output nodes and (b) the information flow from the source to the sink nodes of the network. Here, we describe a novel, scalable, network traversal algorithm and we assess its potential to achieve significant network size reduction in both synthetic and *E. coli* networks. Scalability and sensitivity analysis show that the proposed method scales well with the size of the network, and is robust to noise and missing data.

**Conclusions:**

The method of network flooding proves to be a useful, practical approach towards information flow analysis in gene regulatory networks. Further extension of the proposed theory has the potential to lead in a unifying framework for the simultaneous network minimization and information flow analysis across various “omics” levels.

## Background

In the last decade we have witnessed an explosion of biological data that are available in all branches of the Tree of Life. Significant advances in the biotechnological and computational realm have enabled new ways of acquisition, representation, analysis, and integration of many heterogeneous and seemingly disparate sources of data. This has led to the development of numerous databases that contain validated and putative associations between DNA, proteins or metabolites and various inference algorithms for network reconstruction. Still, in many cases, we lack the methods to extract information that is specific to a cellular mechanism, a biological behavior, or a complex phenotype from the plethora of the available data. The need to develop such methods will become increasingly more obvious, due to the projected accumulation of data in the following years.

Here, we address the problem of network minimization and regulatory information flow in gene regulatory networks. Given a gene regulatory network, a set of source (input) and a set of sink (output) nodes, our task is to find (a) the minimal sub-network that encodes the regulatory program which involves all input and output nodes and (b) the information flow from the source to the sink nodes of the network. Concomitantly, if no sink nodes are specified, our task is reduced to identify the underlying pathways and nodes that are recipients of the information propagated from the source node(s). In this context, source nodes can be thought as cellular components that are sensitive to environmental fluctuations, and they can propagate this information to their downstream targets. In bacteria, this set includes intracellular and transmembrane receptors that participate in complex behaviors, such as chemotaxis and quorum sensing, transcription factors and response-specific proteins that are (in)activated by external environmental stimuli, and sigma factors that initiate system-wide regulatory responses to environmental changes, such as heat shocks and nutrient limitation. In vertebrates, this list expands even more to include proteins involved in signal propagation in the nervous system, tissues, and organs. Similarly, sink nodes represent downstream targets of interest. Some examples include enzymes that participate in metabolic reactions, and proteins that are responsible for complex traits, such as stress-response proteins, motility genes, and genes involved in aerobic respiration.

The theory of network floods that we introduce here is a fundamental extension of network flow theory for networks where (a) interactions can be negative and (b) flow is replicated instead of conserved, as it is the case in regulatory networks. Network flow theory [1-3] has been traditionally applied in other disciplines, including multiprocessor scheduling [4], transportation [5], and sociology [6]. Despite the availability of efficient methods in the field early on [7], network flow theory has not been applied in this biological context. The network flow theory cannot be applied directly to biological systems in general, and gene regulatory systems more specifically, since the interactions that take place in biological networks and the network properties are of different nature when compared to the networks in other applications. One of the most striking difference is that network flow is not conserved in each node, in other words, the sum of all incoming flows is not equal with the sum of all outgoing flows. In fact, most regulatory networks exhibit flow replication, where the sum of the incoming flow is replicated in each of the outgoing interactions. This network characteristic captures the process of transcriptional activation of a gene that itself has multiple downstream targets. In that case, each outgoing regulatory edge from that node can have activatory or inhibitory information that does not have to conform to any flow conservation rule. As an example of this flow replication property, imagine a transcriptional regulator, (e.g. the AraC activator protein) that can bind to two distinct promoters in the genome, which drive two different genes. In that case, upregulation of the *araC* gene will signify the simultaneous up-regulation of the downstream targets (the two distinct genes). In this typical case, there is no conservation rule of any sort, for example the number of AraC copies do not have to be equal to the number of the two gene copies. Furthermore, given saturating levels of regulatory protein, the effect of the regulation will be the simultaneous up-regulation of all upstream genes and thus the *replication* of the information flow to each distinct path. The vast majority of signal transduction network analysis methods are focused on topological features [8], such as motifs and binary interactions between nodes in the network [9]. Other approaches use Boolean theory to infer hidden regulatory pathways [10] or compute the minimal set of nodes that can perform signal transduction independently [11]. Although these methods provide valuable insight, they can’t capture quantitative relationships between nodes that are critical for elucidating the network dynamics, and where the weights of the individual edges have a critical role. In a recent study the information flow of acyclic, activation-only, hierarchical networks was studied using continuous expression models [12]. Other relevant prior work includes the application of elementary modes in signaling and regulatory networks for functional analysis [13], shortest path algorithms for biological interaction paths [14,15], application of Petri-net based analysis to signal transduction pathways [16], partitioning biological data with transitivity clustering [17,18], and measuring information flow through random walks ignoring inhibitory links [19]. In contrast to the methods that mostly target clustering or motif finding in biological data, network flooding can elucidate the regulatory information flow taking into account regulation weight and sign, an important challenge in systems biology [9,20], and perform hypothesis-specific network minimization towards transforming data and networks to knowledge. Although network-based approaches have been developed in the past mainly for metabolic networks [21-23], they are not suitable to be applied in cases where both positive and negative regulation is present and flow conservation does not hold.

**Figure 1:**
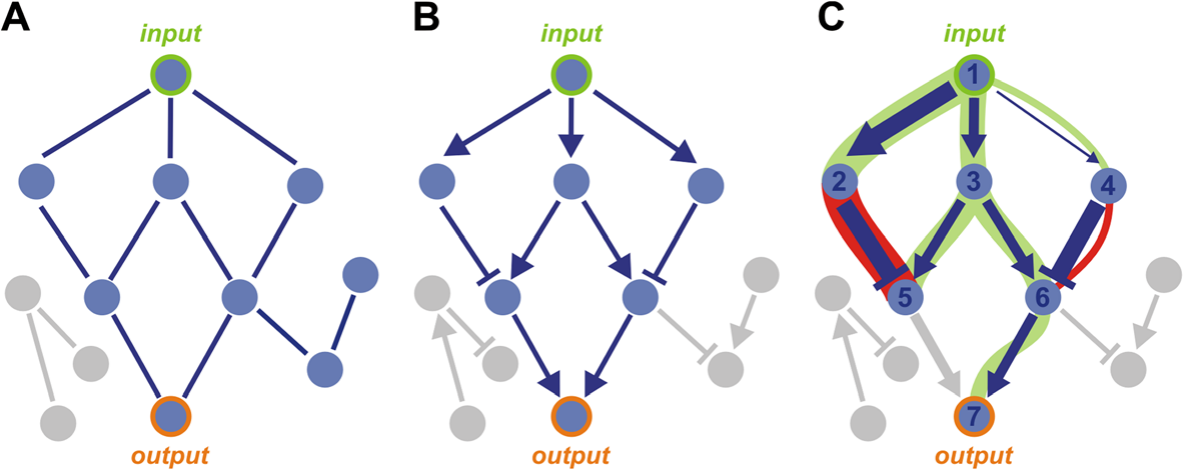
**Flood analysis reveals key pathways in directed weighted regulatory networks. **(**A**) Disconnected nodes can be discarded based on their connectivity to inputs and outputs. (**B**) Direction of links can be used to discard nodes which do not regulate outputs directly or indirectly. (**C**) Flood analysis takes into account weights and signs of directed links and captures up- and down- regulation of nodes. Positive floods from inputs to outputs indicate the pathways that are active for a given scenario (a given set of inputs and outputs), the flood value in each link/node is a metric of that link/node importance.

Figure 1 depicts different scenarios for network minimization and information flow in regulatory networks. Given a set of input/output nodes and an undirected network, node reduction can be achieved by deleting nodes that are not connected to sub-graphs that contain at least one input or output nodes (Figure 1A). In a directed weighted network where regulation follows an additive continuous model where the combined information flow through an edge is a continuous variable and can be less or equal than that edge's weight, this approach can be extended to discard nodes that do not regulate the output node(s) directly or indirectly (Figure 1B). In the flood network analysis that is introduced in this paper, the weight, sign and directionality of the network is taken into account in order to minimize the network and reveal the flow of information in the various pathways (Figure 1C).

Note that network flooding is very different from simple superposition of negative and positive regulatory weights for each node, as it takes into account the amount of regulatory information in the preceding paths. For instance, nodes 5 and 6 have exactly the same regulatory influences (both direction and strength). However, while node 6 will be activated and propagate the signal to downstream nodes, node 5 will be inactive, due to the large information flow from node 1 to node 2 (compared to the small information flow from 1 to 4), which in this case has an inhibitory effect. The outcome of this process is thus highly dependent on both the network topology, and the corresponding weights. The problem becomes more challenging in the presence of loops and cycles, where simple network traversals (such as depth/breadth- first search) are not applicable. In addition, network flooding is fundamentally different from network flows, as it introduces negative regulatory interactions and conservation of flow is not guaranteed for each node, as it would be the case in metabolic processes. The latter is a necessary addition to realistically capture regulatory interactions, as regulatory information is usually replicated as it passes through a node with multiple outputs.

## Methods

In this section, we define the problem of gene regulatory network (GRN) minimization and we introduce the network flooding method along with an algorithmic implementation. Before we describe the method of network flooding, we first define some terms that we will use in its description.

### Definitions

#### Flood networks and network floods

First, we define the *capacity c* (*u, v*) of the edge (*u, v*), between nodes *u* and *v* as the maximum amount of information that can pass from node *u* to node *v*. A *flood network G*  =  (*V*,  *E*) is a directed graph in which every edge (*u, v*) ∈ *E* with *u*, *v*, ∈ *V* has a non-zero capacity *c* (*u, v)* ∈ *R*, and *c *(*u, v*) =0 when (*u, v)* ∉ *E* (i.e. zero capacity where no edge exists between two nodes). In the case of gene regulatory networks, the nodes represent genes and the edges establish the regulatory interactions between them. The capacity of each edge represents the weight and direction of each regulatory interaction, and can be either positive, or negative. We distinguish a *source node s* ∈ *V*, and for simplicity we assume that a path exists from s to all other nodes in *G*.

Since feedback and high interconnectivity is common in gene regulatory networks specifically, and biological networks in general, we have to devise a method to account for all possible walks in a network, which are not necessary simple. For this reason we “unwind” or “traverse” all its walks that are *essential*, which is formally defined as follows:

**Essential walk:** Given a network *G*  =  (*V*,  *E*), a walk *P* = (*x*_1_, *x*_2_…*x*_*n*_) on *G* with (*x*_*i*_, *x*_*j*_) ∊ *E* is *essential*, if between any two successive appearances *x*_*i*_*,x*_*j*_ of any node *v* ∈ *V*, there exists at least one node that does not appear in the walk *P*’ = (*x*_1_, *x*_2_…*x*_*i*_).

An essential walk allows multiple appearances of some nodes, as long as each cycle introduces at least one new node to the essential walk. An essential walk is called *saturated*, if its expansion through any node will make it non-essential. An *essential traversal* of *G*, starting from the source node, is the set *P*^*s*^ of all saturated walks starting from the source node *s*, following a breadth-first manner. For a given edge (*u, v*) we denote as *P*_(*u*,*v*)_^*s*^ = {(*s*, …, *u*, *v*, …)} the subset of all saturated walks within the essential traversal that include the edge (*u, v*). Note that the essential traversal captures feedback loops of arbitrary size. Given the above, the *network flood f* (*u, v*) of an edge (*u, v*) corresponds to the amount of information that flows through that edge, and it can be calculated by any function *f*: *E* → *R*, provided that it is subject to the following three constraints:

*Capacity Constraint:* For all edges (*u, v*) we require that |*f*(*u*, *v*)| ≤ *c*(*u*, *v*)|.

*Polarity Constraint:* For all edges (*u, v*) we require that *f*(*u*, *v*)*c*(*u*, *v*) ≥ 0.

*Essential Walk Constraint:* We denote with *f* (*P*) the flood *f* (*u, v*) to an edge (*u, v*) that is carried through a walk *P* = (*s*, …, *u*, *v*), which starts from the source node *s*. The following constructs by induction a set of essential walks *D*, which defines the essential walk constraint on |*f*(*u*, *v*)|, for all edges (*u, v*): Base case: Initially *D* = ∪ _(*s*,*u*)∊*E*_{(*s*, *u*)} , and we require that |*f*((*s*, *u*))| = |*c*(*s*, *u*)|. Inductive step: Let *P* be a walk in *D*, and *P*′ its expansion through an edge (*u, v*). The essential walk constraint states that the *network flood* in edge (*u, v*) is given by: |*f*(*u*,  *v*)| = |*f*(*P*^'^)|  =   max  (0,   min(|*c*(*u*,  *v*)|, ∑ _*Q* ∈ *D* : *Q* = (*s*,  …  , *w*, *u*)_*f*(*Q*))), with *D* = *D* ∪ {*P*^’^}.

The capacity constraint restricts the magnitude of the flood that can run through an edge. The polarity constraint guaranties that the running flood has the same sign as the edge capacity, to preserve its regulatory function. Finally, the essential walk constraint imposes that each non-saturating walk *P* entering a node has to carry out the same flood as the one it brings into u, and the magnitude of the total flood in any edge (*u, v*) be determined by the algebraic sum of the flood carried by the set $P_{{}^{\left(u,v\right)}}^{s}\;\subseteq \;Q$. In general, the function itself can take any form (e.g. sigmoid, polynomial), similar to the kernel functions in classification, although here we define its magnitude to be the linear sum of all incoming floods from essential walks, with the edge capacity value being its upper bound (capacity constraint), and its sign to be the same as the edge capacity (polarity constraint). The above definitions can be naturally extended to networks with more than one distinguished source nodes.

**Environmental signals and source nodes:** Let *S* = {*s*_1_, *s*_2_…} be a set of continuous variables that encode for environmental signals (e.g. heat, pressure, light, chemicals), each of which can be sensed by the organism through a non-empty set of nodes $\left\{u_{s_{i}}\;\in \;V\right\}$, namely the *receptor* nodes for the corresponding environmental signal. An environmental signal *s*_*j*_ serves as a source node in a flood network, and is defined as “active” when it has a positive value. An edge or a node is defined as “active” when it has a non-zero flood. Floods can be positive or negative, which corresponds to activation or inhibition of the target node (i.e. downstream gene), respectively. Depending on which environmental signals are active, different pathways in the regulatory network are activated as a response to the current environmental state.

#### Network minimization problem

**Figure 2:**
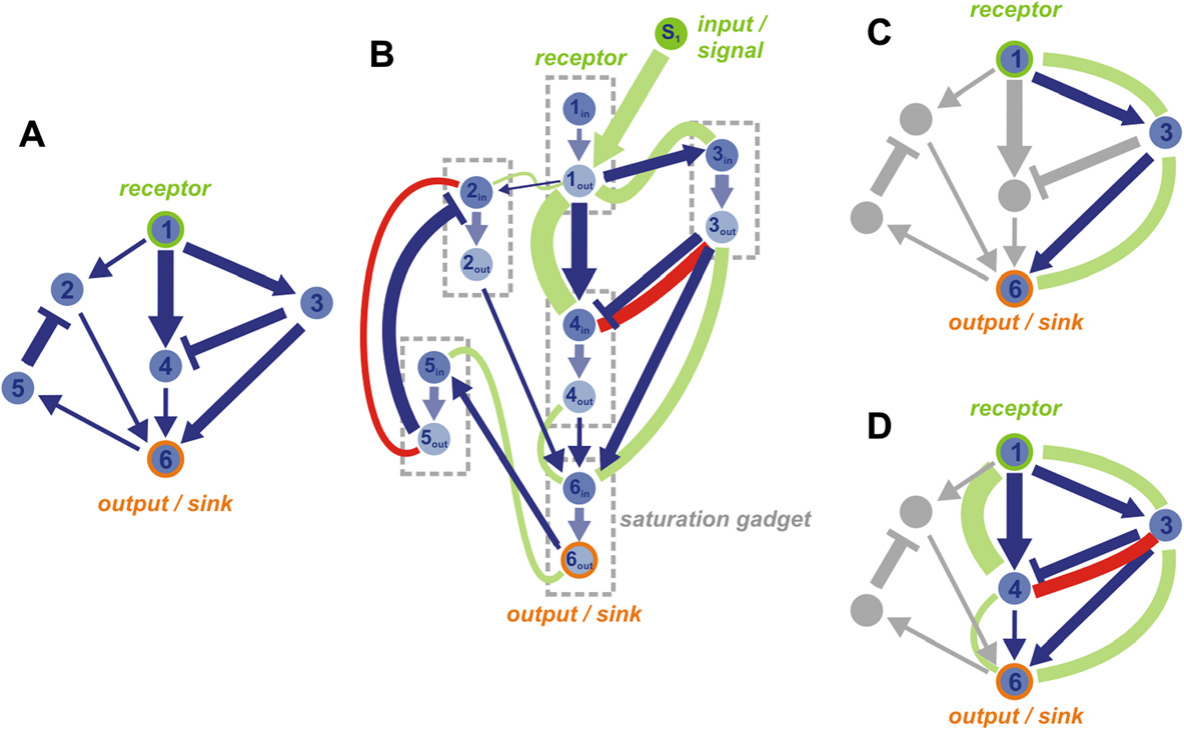
**Example of the *flood network minimization. ***Original *gene regulatory network *(**A**) is *transformed *(**B**) to introduce *saturation gadgets *and potentially *basal nodes* (not shown). Minimized network is obtained after a *flood threshold *is applied to the flooded transformed network. Degree of minimization can be varied from aggressive (**C**) to mild (**D**) depending on the value of the threshold. *Capacity *of the edge is the upper limit for the magnitude of the *flood *through that edge, which depends on the upstream regulation. For example: edge 3→6 has a high capacity and a high positive flood, but the flood through edge 5→2 is low regardless its high capacity. Floods are calculated for the *essential traversals *from the *input(s)/signal(s) *to the *output(s)/sink(s)*: 1→3→6, 1→4→6, 1→4→6→5→2→6, 1→3→6→5→2→6, 1→3→4→6, etc. The *total flood *through edge 2→6 is zero due to the strong negative flood 5→2 (inhibition of node 2). Therefore, nodes 2 and 5 are not part of the minimized network connecting inputs and outputs regardless of the imposed flood threshold. In contrast, node 4 may be included in the minimized network if a flood threshold is relatively low: flood 1→3 is replicated into two edges of high capacity 3→6 (positive flood, activation of node 6) and 3→4 (negative flood, inhibition of node 4). Large positive flood 1→4 in combination with a negative flood 3→4 produces a weak positive flood 4→6. Edge 4→6 is included in the minimized network only if the flood threshold is below 4→6 flood (**D**). If edge 4→6 is not in minimized network (**C**), edges 1→4 and 3→4, and node 4 are also not included, as they are disconnected from the output node(s).

Given a GRN *G* and a set of active environmental signals *S*_*act*_ ⊆ *S*, infer the *minimal GRN G*^’^ = (*V*^’^, *E*^’^), such that *G′* is the sub-graph of *G* that only contains all active nodes *E*′ and active edges *E′*, from the whole set of nodes and edges in *G*. In case that a set of sink nodes *A* ⊆ *V* is also supplied, *E*′ should contain only edges that are found on a path from any nodes *s* ∊ *S*_*act*_ to any nodes *t* ∈ *A*. Figure 2 depicts an example of gene regulatory network minimization problem.

### Regulatory network minimization through flood analysis

**Figure 3:**
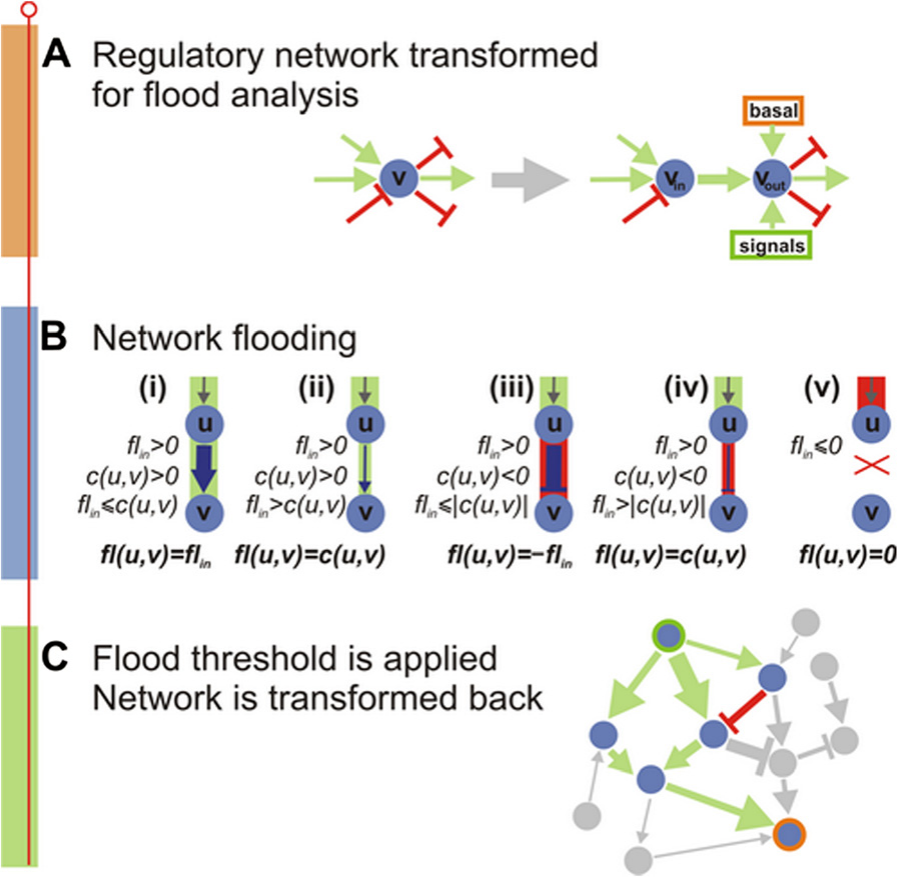
**Pipeline for flooding minimization. **(**A**) Transformation of the regulatory networks for flooding analysis as described in the text. (**B**) Flooding the network; for a given regulator node *u *with a positive total incoming *fl*_*in *_the following scenarios are possible for the flood *fl(u,v) *through the edge *(u,v)*: (i) and (ii) if edge capacity *c(u,v) *is positive and is greater or smaller than the value of the incoming flood, respectively; (iii) and (iv) if edge capacity *c(u,v) *is negative and its absolute value is greater or smaller than the value of the incoming flood, respectively; (v) if the total incoming flood *flood*_*in *_is negative, then the node is down-regulated and no flood is propagated regardless of the capacity of the edge. (**C**) Selection of nodes/links with flood above the threshold and the reverse transformation of the network.

Here, we introduce a three-phase pipeline to address the network minimization problem defined above (Figure 3). Given a GRN G, and a set of active environmental signals S_*act*_, we first transform the input network G to a flood network that captures basic properties of gene networks such as a basal steady-state expression and a saturation limit on the expression of any given gene (phase one). Then, we calculate the floods on the transformed network (stage two). We then perform the actual minimization by imposing a flood threshold and then we inversely transform the resulting network to its GRN counterpart (stage three).

### Network transformation (phase one)

In the first phase, the GRN is transformed to a flood network G=(V,E), which uses signal nodes, basal nodes, saturation nodes, signal and basal regulatory links to capture the basic elements of gene regulation. The steps of the transformation are the following (Figure 3A):

1. Introduction of the *signal nodes.* Every signal ∈ *S*_*act*_ is mapped to a signal node *s* ∈ *V*. The set of all signal nodes serves also as the set of distinguished nodes *S* ⊂ *V*.

2. An additional *basal node b* ∈ *V* is introduced. This node captures the basal (i.e. base) expression of the gene that depends on the "leakiness" of the upstream promoter and the concentration of the transcription factors that are bound to the promoter and regulate the gene's expression.

3. To capture the saturation of the node *v* expression, a *saturation gadget* is introduced*.* For every node *v* in the original GRN, two nodes *v*_*in*_*,v*_*out*_ are introduced together with an edge (*v*_*in*_*,v*_*out*_) the capacity of which *c* (*v*_*in*_*,v*_*out*_), is a positive number that limits the maximum flood through node.

4. Introduction of the *signal* and *basal regulatory links.* For every receptor node *v* in the original GRN regulated by an external signal *s*, (*s*, *v*_*out*_) is added in *E*. The capacity *c*(*s*, *v*_*out*_) is set equal to the corresponding *signal regulation weight w* (*s*, *v*_*out*_) from signal *s* to node *v*. Moreover, in the presence of information on the basal expression levels of a node *v*, an edge (*b, v*_*out*_) is added, with the capacity *c*(*b*, *v*_*out*_) set equal to the basal expression of *v*, *b* (*v*).

The basal node b, serves as an additional source node which is always being active.

### Network flooding (phase two)

In this step we calculate all network floods in the transformed flood network, by applying an essential traversal on it. Algorithm 1 provides computation of floods in a flood network *G* = (*V, E*) with a set of distinct source nodes *S*. The process starts from the nodes in *S*, and is based on repetitive expansions of essential walks until they get saturated. Each walk *P* is carrying some flood, and upon its expansion through an edge (*u, v*), the following take place: (a) the magnitude of the flood to be stored in that edge is determined (*flood*_*total*_) by adding the existing flood on the edge with the one carried by the *P*, (b) possible excess of the incoming flood caused by the saturation limit is stored in a matrix (*Excess*), (c) the flood change (*Flood*_*delta*_) is then propagated along the walk, after being polarized by the sign of the edge. The time complexity of the essential traversal is highly dependent on the network topology and while in the worst case it can scale exponentially with the number of the nodes, scalability analysis shows that our method scales robustly with the increase of the graph size.

## Algorithm 1

### Threshold imposition and reverse network transformation

Now that we have calculated all floods, we can impose a lower bound on the minimal magnitude that we will allow. Conservatively, this value is zero (i.e. even a small amount of information flow is sufficient to add an edge on the minimized network), but any threshold *t* can be used, so that only edges with flood magnitude greater that *t* will exist (i.e. |*f*(*u*, *v*)| >*t*), where negative flood values are also allowed. In case that a set of output (sink) nodes *A* is also supplied, this step additionally disregards edges getting disconnected from A once the threshold is imposed. Transformation the minimized network to its GRN counterpart is achieved by simply reversing the steps performed in phase one. The network flooding method is deterministic for any given threshold.

## Results

Ideally, performance evaluation of the network floods theory requires complete regulatory networks where all nodes and links are present, together with link directionality and a signed weight. In addition, the quantitative expression model for each node should be known, and the phenotypic change after the network reduction should be measurable for informative comparison between the original and minimized network. Since currently we are far from having any such dataset, we first adopted a similar approach to what is used for benchmarking gene network reconstruction algorithms, by constructing synthetic datasets of *in silico* organisms [24]. We used a multi-scale microbial evolution simulator to create a synthetic complete dataset with the information mentioned above to comprehensively evaluate the proposed methods. Our results show that our method has very good scaling, is robust to noise and missing data, and does not require full network knowledge. We then evaluated our method with experimental data in the case of the bacterium *E. coli*, a well-studied model organism*.* The source code, sample data files and a brief tutorial on the network flooding algorithm is provided in Additional file 1.

### Synthetic datasets

We used the EVE (Evolution in Variable Environments) simulator to create a synthetic dataset and applied the network flood algorithm to the resulting networks. The EVE simulator has multiple levels of abstractions that range from molecular species, gene regulatory and biochemical networks, to organisms and environment. Each organism has its own distinct gene regulatory and biochemical network that can be depicted as a directed weighted graph. The network comprises of a number of “triplets” (three nodes): Gene/mRNA, Protein, and Modified Protein. The Promoter/Gene/RNA node captures gene regulation and transcription, while the Protein and Modified Protein nodes capture translation and post-translational modification (acetylation, phosphorylation, etc.), respectively. In other words, the triplets capture the “central dogma” of molecular biology. Organisms undergo a stochastic evolution and their gene regulatory and biochemical networks change in size and topology in order to adapt to the synthetic environments. EVE has been used to test the hypothesis of anticipatory behavior in bacteria [25], to investigate Horizontal Gene Transfer dynamics [26], the distribution of fitness effects during evolution [27], facilitated variation in microbial communities [28], and has been documented elsewhere [29]. Here, 64 populations of 256 organisms each where evolved in low and high mutation rates (lmr/hmr) for 5,000 generations in dynamical environments where the existence of two environmental signals and the presence of nutrients follows a either an AND or a XOR gate (more about environmental structure in the supplementary online material of [25]). This resulted on a dataset of 47,698 organisms (after the complete set was filtered for organisms of high fitness) evolved in a total of four environments with complete information on their network connectivity, kinetic parameters, expression, evolutionary trajectory, and fitness.

As an exhaustive search has a Θ (2^*m*^) complexity for a network of m links and thus is infeasible to run, we compare our method to the heuristic available that achieves reduction close to that of exhaustive search [25]. Table 1 shows the efficiency of network flooding in terms of running time (up to 10^4^ speed-up) and link removal in the final networks.

**Table 1:** Mean network minimization and mean running time comparison in synthetic datasets

	**Mean initial size, links**	**Mean minimization, % of removed links**		**Mean fitness decrease in flood minimized networks**	**Mean running time, sec**	
		**Flood**	**Best heuristic**		**Flood**	**Best heuristic**
AND-lmr	11.61	-52.3	-66.9	-0.8%	0.0019	11502.0
AND-hmr	26.57	-41.1	-83.3	-1.0%	0.0023	12147.0
XOR-lmr	52.20	-34.0	-79.7	-1.9%	0.0036	13794.0
XOR-hmr	55.68	-33.9	-81.1	-2.0%	0.0039	13986.0

**Figure 4:**
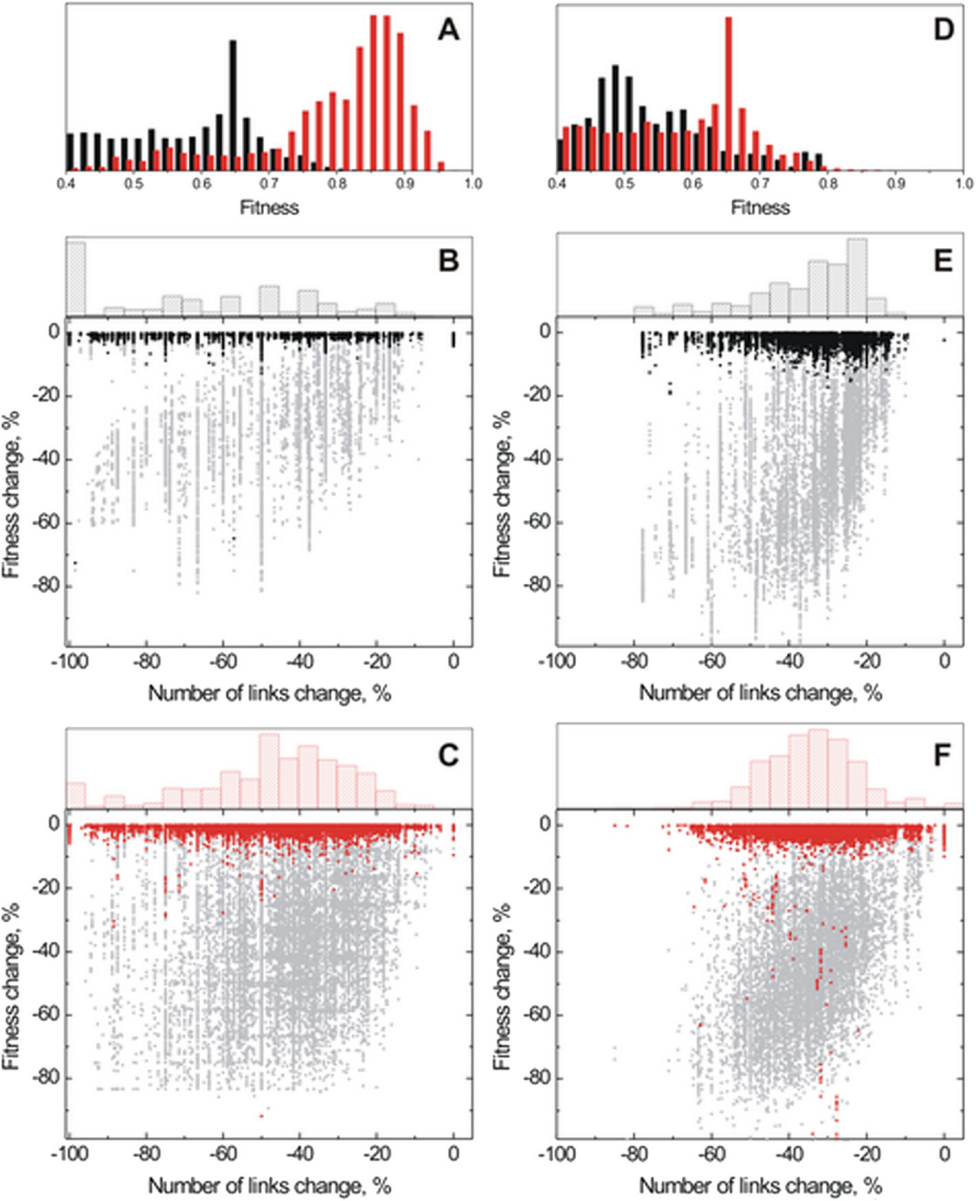
**Flood-based minimization of regulatory networks of *in silico *organisms evolved in AND (A-C) and XOR (D-F) environments. **Top panels (**A **and **D**) show the distribution of fitness for cells evolved in high mutation rates (red) and low mutation rates (black). Dot plots show the statistics of the flood minimization for populations of cells evolved in AND low mutation rate (**B**), AND high mutation rate (**C**), XOR low mutation rate (**E**), and XOR high mutation rate (**F**) environments. Gray dots show the effect on fitness of a random network minimization to the same degree as obtained by the flood analysis. Bar plots in (**B**, **C**, **E**, and **F**) show the distribution of minimization degree (decrease in number of links) for each type of evolved cells.

Interestingly, our analysis shows that application of network flooding to network minimization can highly reduce the number of links with a minimal impact to fitness (Figure 4). In the case of XOR, the average reduction of evolved cells is 33% and 34% of links for low and high mutation environment, respectively, and the average change of fitness is -1.9% and -2.0%, as shown in Table 1. In contrast, a random deletion of the same percentage of links leads to deleterious changes in the gene network and major phenotypic change (grey dots, Figure 4 and Table 1). This result is even more pronounced for the case of the AND environment. Decrease in fitness by random removal of links is expected, as a subset of these links is likely to be important for the organism to exhibit the desired phenotype (XOR and AND, respectively). Additionally, cells that evolved in environments with higher mutation rates, contained cells that exhibited higher stochasticity in their expression levels, and the network flooding resulted in a larger fitness loss, but still for <0.1% of cells. Similar results have been found for cells that have been evolved in an OR environment (31129 cells; Additional file 2: Figure S1).

### Scalability and sensitivity analysis

**Figure 5:**
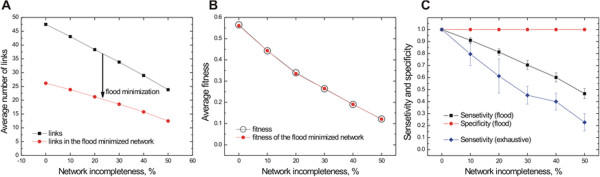
**Effect of network incompleteness on the performance of network minimization in the XOR environment. **(**A**) The average size of links is reduced linearly but with decreased slope, (**B**) the minimized and initial network (after random link removal) have the same fitness, (**C**) the sensitivity of the method (how many of the true positive links are present in the minimal network is on par or better to that of the heuristic search. Analysis performed in sets of 100 cells over 10 randomizations. Similar statistics were gathered in the case of the AND environment (Additional file 1).

We evaluated the scalability of our algorithm in respect to node size, and it was found to scale well below the exponential theoretical upper bound (Additional file 2: Figure S2). To assess the performance of our method when data are missing, we randomly removed sets of links from the network before minimization (Figure 5 and Additional file 2: Figure S3). This analysis is necessary as even for *E. coli*, the amount of missing information is high as it currently has a reconstructed network of about 1700 genes (38% of the genome) and many missing associations. Our method was found to be robust even in cases where half of the biological network is unknown. Similarly, perturbation of 10% of the weight value did not affect the network minimization result (<1% fitness change).

#### *Escherichia coli* regulatory network

**Figure 6:**
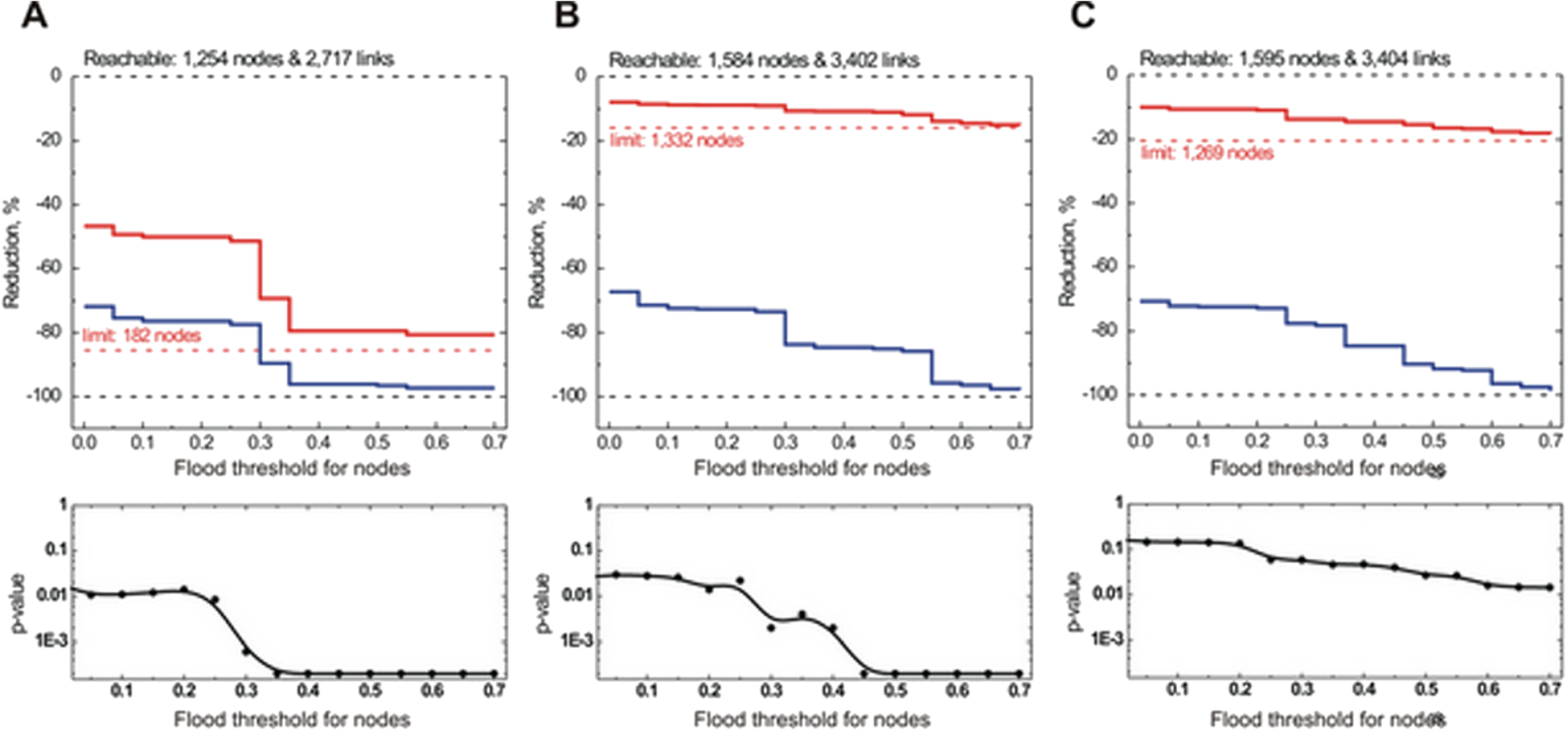
**Flood minimization of the *E. coli *gene regulatory network for different scenarios. **(**A**) Stationary phase scenario, where the source node is sigma factor σ^38^ and reporter nodes are genes that were found to be over expressed in the stationary phase in transcriptional profiling experiments. (**B**) Exponential growth scenario, where input signals are the sigma factors σ^70^, σ^38 ^and σ^54^; reporter nodes are genes with Gene Ontology annotation related to the exponential cell growth. (**C**) Transition phase scenario, where input signals are the σ^70 ^and σ^38^ sigma factors; reporter nodes are the top genes expressed during the transition from exponential to the stationary phase. Solid and dash-dot lines depict link and node removal, respectively; p-value is calculated by the hypergeometric distribution with multiple hypothesis testing correction (Bonferroni); “flood threshold” refers to the threshold *t *in the methods section; “reachable” network refers to the complete sub-network of nodes that is connected, directly or indirectly, to the sigma factors that are active in the corresponding condition (both topology and directionality is used to extract the reachable network); reduction is shown as a percentage of the reachable network.

**Figure 7:**
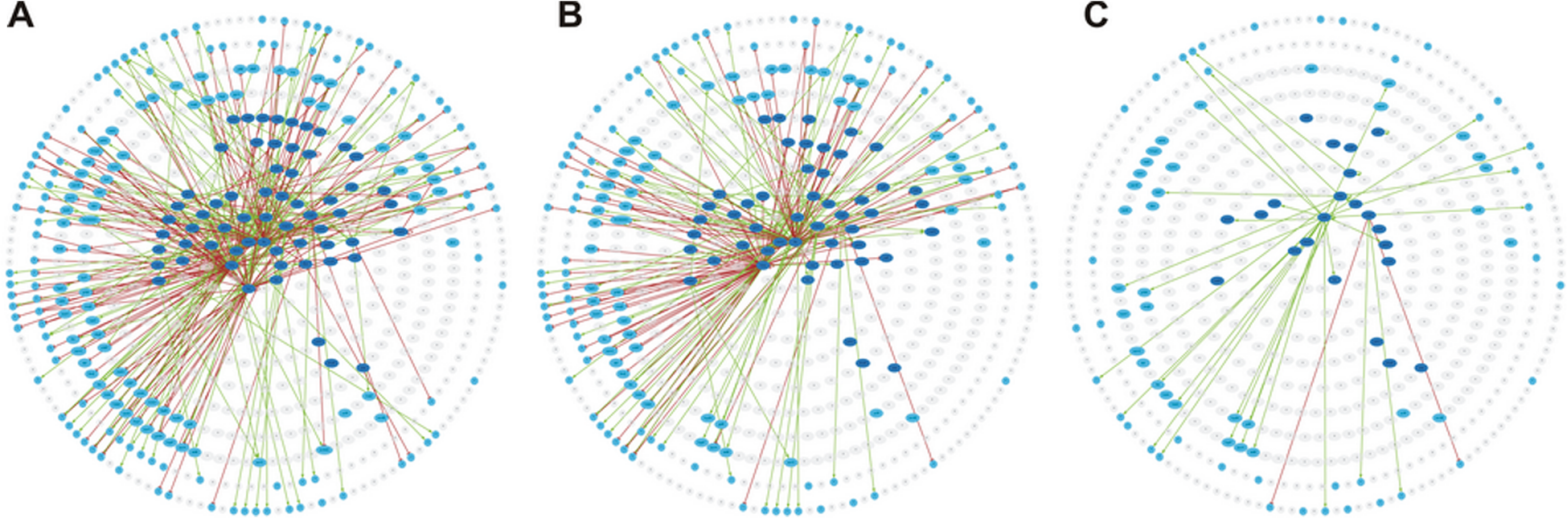
**Flood network minimization overview for the *E. coli *gene regulatory network in the “Stationary phase” scenario.** (**A**) Sub-network “reachable” from the inputs, which contains all nodes directly or indirectly regulated by the input nodes; (**B**) and (**C**) reduced sub-networks of nodes with the flood above 0.25 and 0.65 thresholds, respectively. Nodes represent genes; dark blue nodes in the center are the regulator nodes; light blue nodes in the outer circles are the regulated nodes (regulated genes from the same transcription unit and identical regulation are grouped together); grey nodes are the genes which are not included into the sub-network.

Next, we reconstructed the regulatory network of *E. coli* from data available in EcoCyc [30] and RegulonDB [31]. Compared to the networks in the synthetic dataset, the derived *E. coli* network is incomplete with many genes measured in different environmental conditions and of unknown function or regulation (more than 1000 genes are not connected to any sigma factor). Unit regulatory weights were used in this example and Gaussian noise with a standard deviation of 0.05 was added. To evaluate our network flooding algorithm, we considered several scenarios that are relevant to *E. coli* growth and stress response. We used sigma factors as information source nodes, as they act as master regulators. under various scenarios and their relative concentration ratios are known for each of the conditions we consider here [32]. To assess the performance of our algorithm, we created a set of reporter genes for each condition that are likely to be involved in the respective processes. This set includes genes that have been differentially expressed in these conditions (microarray data provided in [33]), and are implicated in cellular response as indicated by their GO terms. In this context, our network flooding analysis has been used to reveal regulatory information flow, and perform network minimization (Figure 6). A functional analysis of the genes in the minimized network show consistent patterns with what is biologically known for growth in the conditions that we focus on (See Additional file 2: Tables S1-3). More specifically, under “exponential growth” (Figure 6B and Table 2) among the over-represented terms where protein complex (p-value 2.7 10^-12^, GO:0043234), cellular respiration (2.6 10^-9^, GO:0045333), chemotaxis (2.2 10^-8^, GO:0006935), generation of precursor metabolites and energy (7.9 10^-8^, GO:0006091), carbohydrate transport (1.3 10^-7^, GO:0008643), membrane part (1.110^-6^, GO:0044425), amino acid transport (2.7 10^-6^, GO:0006865). Under the stationary phase scenario (Figure 6A and Table 2), over-represented terms include oxidative phosphorylation (5 10^-7^, GO:0006119), anaerobic respiration (2.610^-7^, GO:0009061), oxidation-reduction process (5.4 10^-5^, GO:0055114), nitrogen utilization (2.910^-5^, GO:0019740), carboxylic acid transport (3.4 10^-4^, GO:0046942). Table 2 summarizes the different scenarios that we consider, along with the sizes and average floods in each case. Network flooding is able to minimize the network, while still preserving statistical significance of the results (Figure 6). P-values can be viewed as the probability that the reporter nodes obtained in the minimized network are by random chance. Figure 7 depicts the minimal network for different flood thresholds.

**Table 2:** **Scenarios used in the flood minimization of *E. coli *regulatory network**

**Scenario**	**Inputs**	**Reporter gene selection**	**Genes in a sub-network**	**Reporter genes**	**Total flood in a network with the flood threshold equals to**		
					0.00	0.35	0.70
Stationary phase	σ^38^	Stationary phase specific genes under control of σ^38^ (22)	1,254	10	185	50	33
Exponential growth / GO groups	σ^70^, σ^54^, σ^28^	Genes based on GO terms (amino acid synthesis, translation, protein folding, protein modification, glycolysis, tricarboxylic acid cylce)	1,584	168	242	49	48
Exponential growth / expression data	σ^70^, σ^54^, σ^28^	Genes expressed in the exponential phase (microarray expression data (23)	1,584	54	232	96	40
Heat shock / GO groups	σ^38^, σ^54^, σ^32^	Genes expressed in the exponential phase (microarray expression data (23)	1,314	13	173	46	33
Transition phase / expression data	σ^70^, σ^38^	Genes expressed in the transition from the exponential to the stationary phase (microarray expression data (23)	1,595	34	241	102	52

Total number of genes is shown for a sub-network regulated by the selected inputs. Total flood is shown for full and minimized networks with three different flood thresholds (the maximum flood through a link is 1.0).

## Conclusions

In this paper, we have presented the method of network flooding that aims to minimize regulatory networks in order to capture core regulatory patterns and information flow for specific biological conditions. We introduced a scalable, robust, graph-based algorithmic implementation that can achieve impressive network size reduction, without disrupting core regulatory pathways in synthetic datasets. When network flooding was applied in the reconstructed E. coli regulatory network, it was able to reduce its size producing meaningful (in terms of biological processes involved) and statistical significant (in terms of differentially expressed genes and GO terms) results. In addition, network topology is sufficient for network flooding to operate at the lack of other data sources, and the method copes well with missing information and unknown relationships. There are numerous extensions to our work that can prove useful for biological network analysis and pathway extraction. The presented method can be used to ask questions regarding the maximum information and (regulatory) control that can be achieved by any given node or set of nodes; the importance of a single node or sub-network manifested by the amount of information flow that it channels, which is a quite different metric than its connectivity or regulatory strength; and the degree of multiplexing information that can be achieved in various organisms, a possible proxy for regulatory complexity. We intend to apply the method of network flooding in reconstructed mammalian networks, both in respect to regulation of core mechanisms [9], and miRNA-based regulation [15]. Although we have mainly focused so far on regulatory networks, this work can be extended in protein-protein interaction (PPI), signal transduction and metabolic networks. This entails the extension of the network flood theory in order to handle differently nodes and edges that belong to distinct network types, as the associations between nodes are usually different (for example, a link between two nodes in a metabolic network usually signifies conversion, and not regulation). Taking into account the high degree of interconnection between multiple scales of biological organization, this extension may lead to a unifying framework for the simultaneous network minimization and information flow analysis across various “omics” levels, that is more than the sum of its parts.

## Abbreviations

GRN: Gene regulatory network; GO: Gene ontology; PPI: Protein-protein interaction; lmr: Low mutation rate; hmr: High mutation rate.

## Competing interests

The authors declare that they have no competing interests.

## Authors’ contributions

Corresponding author IT conceived the project and supervised all its aspects; AP designed the algorithms and performed the experiments; IT, AP and VM analyzed the data and wrote the manuscript. All authors read and approved the final manuscript.

## Supplementary Material

Additional file 1 The source code, samples and a brief tutorial for NetFloods. Click here for file

Additional file 2 Supplementary text, figures and tables. Click here for file
