# Supplementary material for: A flood-based information flow analysis and network minimization method for gene regulatory networks
Source: BMC Bioinformatics. 2013 Apr 24;14:137. doi: 10.1186/1471-2105-14-137 (PMC3672003; doi:10.1186/1471-2105-14-137)
Supplement: Additional file 1 — The source code, samples and a brief tutorial for NetFloods. [file 1471-2105-14-137-S1.zip › NetFloods/readme.pdf]

NetFloods 1.0

5/14/2012

NetFloods code is an implementation of the network flooding theory introduced in [1]. It aims to address the problem of network minimization and regulatory information flow in biological networks. Given a regulatory biological network, and a set of source (input) and sink (output) nodes, the task is to find (a) the minimal sub-network that encodes the regulatory program involving all input nodes and (b) the information flow from the source to the sink nodes of the network. A novel network traversal algorithm is capable of significant network size reduction in both synthetic and biological networks without disrupting the core regulatory pathways.

1. Installing NetFloods

The code is written in Python; version 2.7.x is required and can be obtained at http://www.python.org/download/. Python comes preinstalled on most Linux-based systems, however it needs to be installed manually in MS Windows.

NetFloods does not require any installation, but the following Python libraries need to be installed:

> NumPy (http://numpy.scipy.org/) – most major Linux distributions currently come with NumPy preinstalled, otherwise the library can be obtained by most standard package managers or compiled from the source. Binary installation is available for MS Windows and Mac OS X.
>
> NetworkX (http://networkx.lanl.gov/download.html) – the library can be obtained by most standard package managers in Linux-based systems or compiled from the source under other operating systems.

2. Running NetFloods

The two main programs are: (1) flood_minimize.py to minimize networks based on the flood from the input nodes and (2) flood_analyze.py flood minimizes the network using a series of thresholds; collects statistics for a given set of reporter nodes by repeating minimization using repeated network transformations, the parameters of which are subject to 10% uniform noise on the default parameters. Both programs can be executed by running: `python flood_X.py <parameters>`.

The following parameters are used in both codes:

`-h, --help` show this help message and exit

`-n SOURCE_NETWORK, --source=SOURCE_NETWORK`
  Full input network; default value: "samples/ecoli.network"

`-s SIGNALS_FILENAME, --signals_network=SIGNALS_FILENAME`
  Signals file that contains the signal-node connectivity; default value: "samples/sigmafactors_all.network"

`-t FLOOD_THRESHOLD, --threshold=FLOOD_THRESHOLD`
  In flood_minimize.py a flood threshold; in flood_analyze.py a comma separated list of flood thresholds. An edge is considered to be active if its flood is above the threshold; default value: "0.0" and "0.0,0.25,0.5,0.75" for flood_minimize.py and flood_analyze.py, respectively.

`-o OUTPUT_NETWORK, --output=OUTPUT_NETWORK`
  Output minimized network file name or output file name prefix in flood_minimize.py or flood_analyze.py, respectively; default value: "ecoli_minimized.network"

`-l LOG_FILENAME, --log=LOG_FILENAME`
  Output log file that stores the parameters of the flooding run; default value: "LOG"

In addition `flood_minimize.py` has the following parameter:

`-f FLOOD_OUTPUT_NETWORK, --flood_output=FLOOD_OUTPUT_NETWORK`
Output full network with flood for each edge; default value: "`ecoli_flood.network`"

And `flood_analyze.py` has the following additional parameters:

`-r REPORTER_NODES, --reporter_nodes=REPORTER_NODES`
File with the list of reporter nodes

`-p REPEAT_TIMES, --repeat_times=REPEAT_TIMES`
Repeat minimization for `REPEAT_TIMES` times using different random network transformations. If >1 then statistics are collected for a given set of reporter nodes; default value: "`1`"

3. Examples

3.1 Minimize *E. coli* network using all known sigma factor's connections as inputs nodes:

```
python flood_minimize.py –n samples/ecoli.network –s samples/sigmafactors_all.network
            -t 0.2 -l logfile.txt
```

Output file `ecoli_minimized.network` contains the minimized network which includes only edges with flood above the threshold (0.2), output file `ecoli_flood.network` contains full initial threshold with flood through all edges.

3.2 Minimize *E. coli* network using a set of sigma factors from a "heat" scenario for a series of thresholds, collect statistics for the reporter nodes for a "heat scenario":

```
python flood_analyze.py -p 10 -s samples/heat.network -r samples/heat.csv -o output
```

A pair of output files (minimized and flood networks) with prefix "`output`" is created for four thresholds (0.0, 0.25, 0.5, and 0.75). p-value statistics is reported in output_parameter_sweep.txt file. Log is saved in the default LOG file.

3.3 Five scenarios used in [1] to minimize and analyze the *E. coli* gene regulatory network:

```
python flood_analyze.py -p 1000 -s samples/exponential.network -r samples/exponential.csv
-o exponential –t 0.0,0.05,0.1,0.15,0.2,0.25,0.3,0.35,0.4,0.45,0.5,0.55,0.6,0.65,0.7

python flood_analyze.py -p 1000 -s samples/exponential.network -r samples/
exponential_extended.csv -o exponential_extended –t
0.0,0.05,0.1,0.15,0.2,0.25,0.3,0.35,0.4,0.45,0.5,0.55,0.6,0.65,0.7

python flood_analyze.py -p 1000 -s samples/stationary.network -r samples/ stationary.csv -
o stationary –t 0.0,0.05,0.1,0.15,0.2,0.25,0.3,0.35,0.4,0.45,0.5,0.55,0.6,0.65,0.7

python flood_analyze.py -p 1000 -s samples/ transition.network -r samples/transition.csv
-o transition –t 0.0,0.05,0.1,0.15,0.2,0.25,0.3,0.35,0.4,0.45,0.5,0.55,0.6,0.65,0.7

python flood_analyze.py -p 1000 -s samples/heat.network -r samples/heat.csv -o heat –t
0.0,0.05,0.1,0.15,0.2,0.25,0.3,0.35,0.4,0.45,0.5,0.55,0.6,0.65,0.7
```

4. Input and output files

Network (input, `-n` option and output, –o option)
Three column, space separated file. One line per network edge. First column contains the source node of an edge, the second column contains the sink node of that edge, and the third column the weight of the edge. Any additional columns are ignored during parsing. Examples: `samples/ecoli.network` and `samples/ecoli_minimized.network`.

Signals (input, `-s` option)
Signal connectivity in the network format. Here source nodes are unique names for input signals and sink nodes are the nodes of the input networks. The edge weights for signal connectivity is ignored. Example: `samples/heat.network`.

Reporter nodes (input, `-r` option)
Two column, comma separated table (csv). A row per reporter node; first column an optional tag (e.g. a GO term), second column is the reporter node name (use the same name convention as in the input network file). Example: `samples/heat.csv`.

Minimized network with floods (output, `-f` option)
Network flood output is similar to network format, but it contains an extra column indicating the flood value for each edge. Example: `samples/ecoli_flood.network`.

Parameter sweep file <prefix>_parameter_sweep.txt (output, only for `flood_analyze.py` with –p 2 or greater)
Statistics for reporter nodes over various random network transformations for a given set of reporter nodes over a given set of flood thresholds. Example: `samples/heat_p_parameter_sweep.txt`.

5. The structure of the code

The code consists of the following modules:

- `flood_minimize.py` executes flood minimization on a given network using a set of input signals
- `flood_analyze.py` applies flood minimization and collects evaluation statistics based on a set of reporter nodes (outputs)
- `flood_minimize_parser.py` command line interface for `flood_minimize.py`
- `flood_analyze_parser.py` command line interface for `flood_analyze.py`
- `make_network.py`: loads a network from a file
- `flood_network.py`: the pipeline functions related to transforming and flooding a network
- `functions.py`: functions for scheduling various flooding experiments and performance evaluations
- `parameters.py`: defines various internal parameters for network construction and flooding

References

[1] Pavlogiannis A, Mozhayskiy V and Tagkopoulos I. A flood-based information flow analysis and network minimization method for bacterial systems, *submitted* (2012).
