# Supplementary material for: A flood-based information flow analysis and network minimization method for gene regulatory networks
Source: BMC Bioinformatics. 2013 Apr 24;14:137. doi: 10.1186/1471-2105-14-137 (PMC3672003; doi:10.1186/1471-2105-14-137)
Supplement: Additional file 2 — Supplementary text, figures and tables. [file 1471-2105-14-137-S2.docx]

**Supplementary Online Material**

**A flood-based information flow analysis and network minimization method for bacterial systems**

Andreas Pavlogiannis, Vadim Mozhayskiy, and Ilias Tagkopoulos*

^*^Corresponding author: Ilias Tagkopoulos, Department of Computer Science and Genome Center, University of California, Davis, Davis 95616, USA. E-mail: [itagkopoulos@ucdavis.edu](mailto:itagkopoulos@ucdavis.edu). Phone: (530) 752-7707

**1. Complexity of the essential walk expansion algorithm**

Estimating the time complexity of the essential walk expansion requires a novel combinatorial approach and is highly dependent on the topology of the graph G=(V,E), rather than the network size. In the following we provide big-O notations for specific types of graphs.

**1.1 Graphs without cycles**

**Lemma:** The total number of times that the essential walk expansion touches all the nodes is at most $2^{n-1}$, with n the number of nodes.

*Proof:* Since no cycle exists, any possible walk expansion is non-saturating, preserving the essentiality of the walk. Apply a topological ordering on the nodes$P=(v_{0}=s,v_{1},\ldots v_{n-1})$.Then any node $v_{i}$, $i>0$, will be touched at most ${t\left( v_{i} \right)=2}^{i-1}$ times, while trivially $t\left( v_{0} \right)=1$. The proof of this statement goes by generalized induction on this ordering:

- *Base case:* The lemma holds trivially for $v_{1}$ which must have exactly one incoming edge $(v_{0},v_{1})$.
- *Inductive step:* Assume the lemma holds for all nodes up to $v_{i-1}$. Because of the topological ordering,$\forall\left( v_{j},v_{i} \right)\in E, j\leq i-1$.Then $t\left( v_{i} \right)=\sum_{\left( v_{j},v_{i} \right)\in E} t\left( v_{j} \right)\leq t\left( v_{0} \right)+\sum_{k=1}^{i-1} 2^{k-1}=1+2^{i-1}+1=2^{i-1}$.


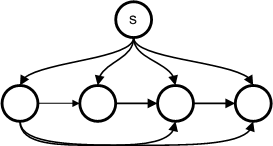
Then, the total number of times that any node is touched is $\sum_{i} t\left( v_{i} \right)\leq2^{n-1}$. This occurs by summing the result of the induction for each node, and can alternatively be visualized by adding an auxiliary sink node t to the graph with incoming edges from any existing node. Then t sums all the visits to every other node in the graph, and by the previous lemma this sum is bounded by$2^{n-1}$. The inductive proof above serves as a structural induction for the worst case graph construction, concluding that the running time of the essential walk expansion is $\Theta\left( 2^{n} \right).$

**1.2 Graphs with non-overlapping cycles**

A graph has no overlapping cycles if no pair of cycles $c_{1},c_{2}$ exists such that $c_{1}$ and $c_{2}$ share a common node. Assume that G has k non-overlapping cycles.

**Remark:** Any node v can be visited from the same walk at most twice. Indeed, assume for the sake of the contradiction that exists a walk P in which v appears at least 3 times. By definition, P will have a subwalk p of the form $p=(v,\ldots,u,\ldots,v,\ldots w,\ldots,v)$, in which w appears for the first time. Then $c_{1}=v,\ldots,u,\ldots,v$ and $c_{2}=v,\ldots w,\ldots,v$ are different, overlapping cycles, since w appears only in one of them (different), with v the common node (overlapping).

Since there are no overlapping cycles, consider a version of G, G’ of size m, in which every cycle has been contracted to a single node, and apply a topological ordering in G’. This will give an ordering of the circles, $C=(c_{0},c_{1},.. c_{k-1})$.Examine any node v appearing in a cycle $c_{i}$, which is touched by m walks that do not traverse $c_{i}$. Because of the above remark, v can be touched at most 2m times overall. Applying the previous case on G’ guarantees that each node v will be visited $t'\left( v \right)\leq2^{m}$ times, $m<n-2k.$

Now, iteratively expand every cycle $c_{i}$, and let $n_{i}$ and $l_{i}$ denote the number of nodes in $c_{i}$ and the number of actual incoming links, respectively.

**Lemma:** For any such expansion, $\forall v\in G,t(v)$ is increased by at most a factor of $2l_{i}n_{i}$.

*Proof:* Indeed, since $c_{i}$ has ­$l_{i}$ incoming edges, any node w in $c_{i}$ can be visited at most $l_{i}$ additional times, *without traversing* $c_{i}$*.* Because of the previous remark, allowing for walks that traverse $c_{i}$ will, at most, double the total visits on w. Summing for every $w\in c_{i}$, at most $2l_{i}n_{i}$ new walks are created from this expansion, which, because of the topological ordering on G’ will be directed towards that part of G’ that has no expanded cycles. In the worst case all of them will go through all the remaining nodes, thus $\forall v\in G,t(v)$ is increased by at most a factor of $2l_{i}n_{i}$.

Thus, after completing the cycle expansion process, each $v\in G$ will be visited at most ${t'\left( v \right)2}^{k}\prod_{c_{i}} l_{i}n_{i}.$ Observe that because of the non-overlapping cycles,$\sum_{c_{i}} l_{i}+n_{i}\leq2n$, thus $\prod_{c_{i}} l_{i}n_{i}.\leq\left( \frac{2n}{k} \right)^{k}$ to conclude that $t\left( v \right)\leq2^{n-k}\left( \frac{2n}{k} \right)^{k}$. Summing for all v, the complexity of the essential walk expansion is $O(n2^{n}\left( \frac{n}{k} \right)^{k})$.

**2. Testing for essential walks**

Let $G=(V,E)$ be a flood network, $\left| V \right|=n, \left| E \right|=m$. An essential walk can be exponentially long with respect to $m$, which implies the need for exponential space if we are to store the walk itself as a sequence of visited nodes. We will describe a method for unraveling essential walks, using linear space in the size of the network, or constant space in the size of the walk. This is possible because testing if a potential walk expansion leads to a non-essential walk can be done without storing the complete history of the walk. The algorithm follows:

**For every walk P, maintain the following data structures:**

- *last_discovered:*A variable that contains the last link that has been discovered and added for the first time in P.
- *link_last_discovered:* An m-size vector. *link_last_discovered[i]* contains the value of *last_discovered*when link i was traversed for the last time from P.

**Testing the expansion of a walk P through a link j is essential:**

- if*link_last_discovered[j] == last_discovered* then the expansion is non-essential, otherwise it is essential.

If *link_last_discovered[j] == last_discovered* then between the last and the current traversal of j, no new links have been visited for the first time, thus expanding P with j will turn it to non-essential. On the other hand, if *link_last_discovered[j] != last_discovered* then a new link has been added in P, and thus P can be expanded through j without violating its essential property.

**Supplementary Figures**


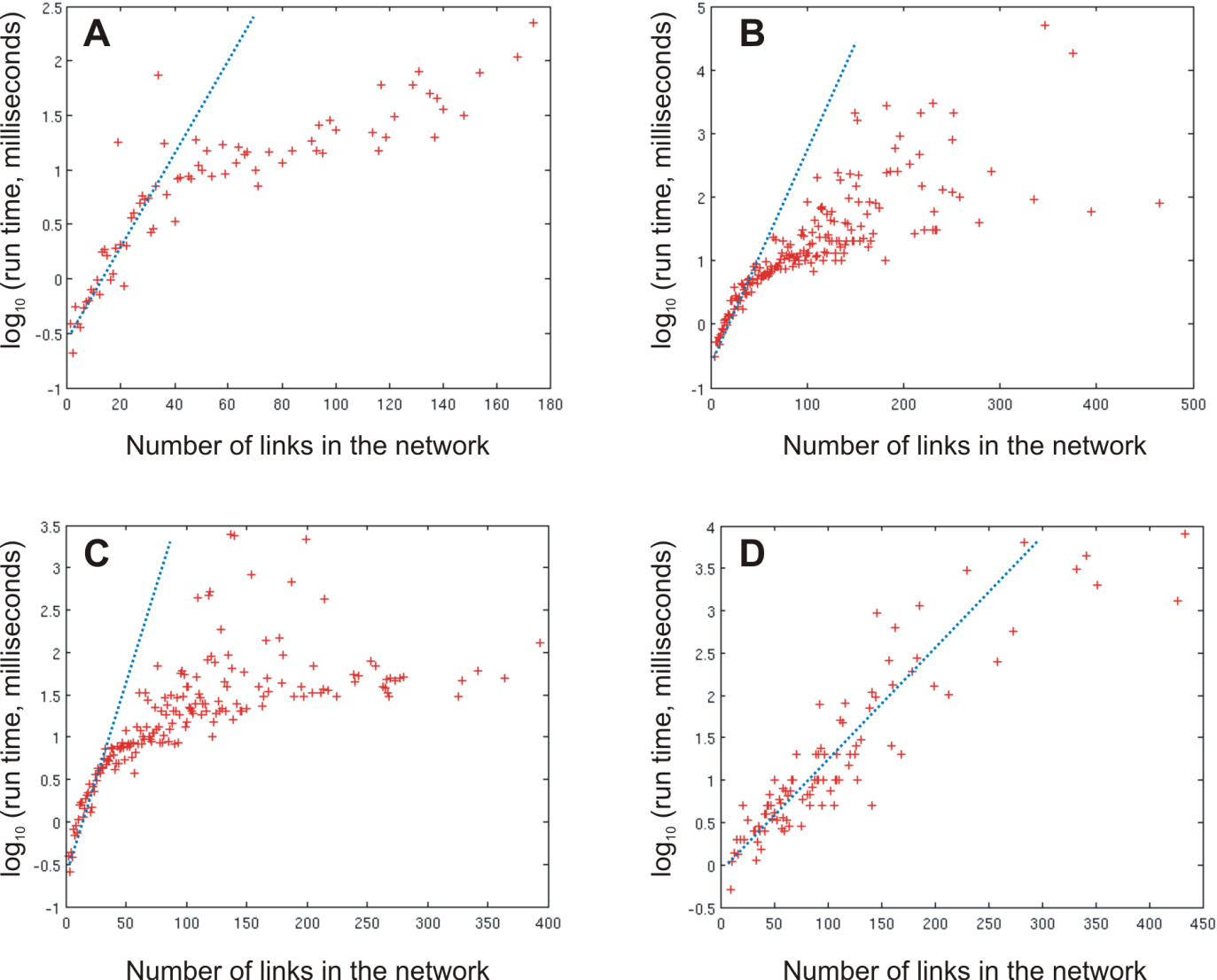


**Figure S1.** Scalability analysis for the synthetic populations evolved in an AND environment under the low and high mutation rates (A and B, respectively) and in an XOR environment under the low and high mutation rates (C and D, respectively).


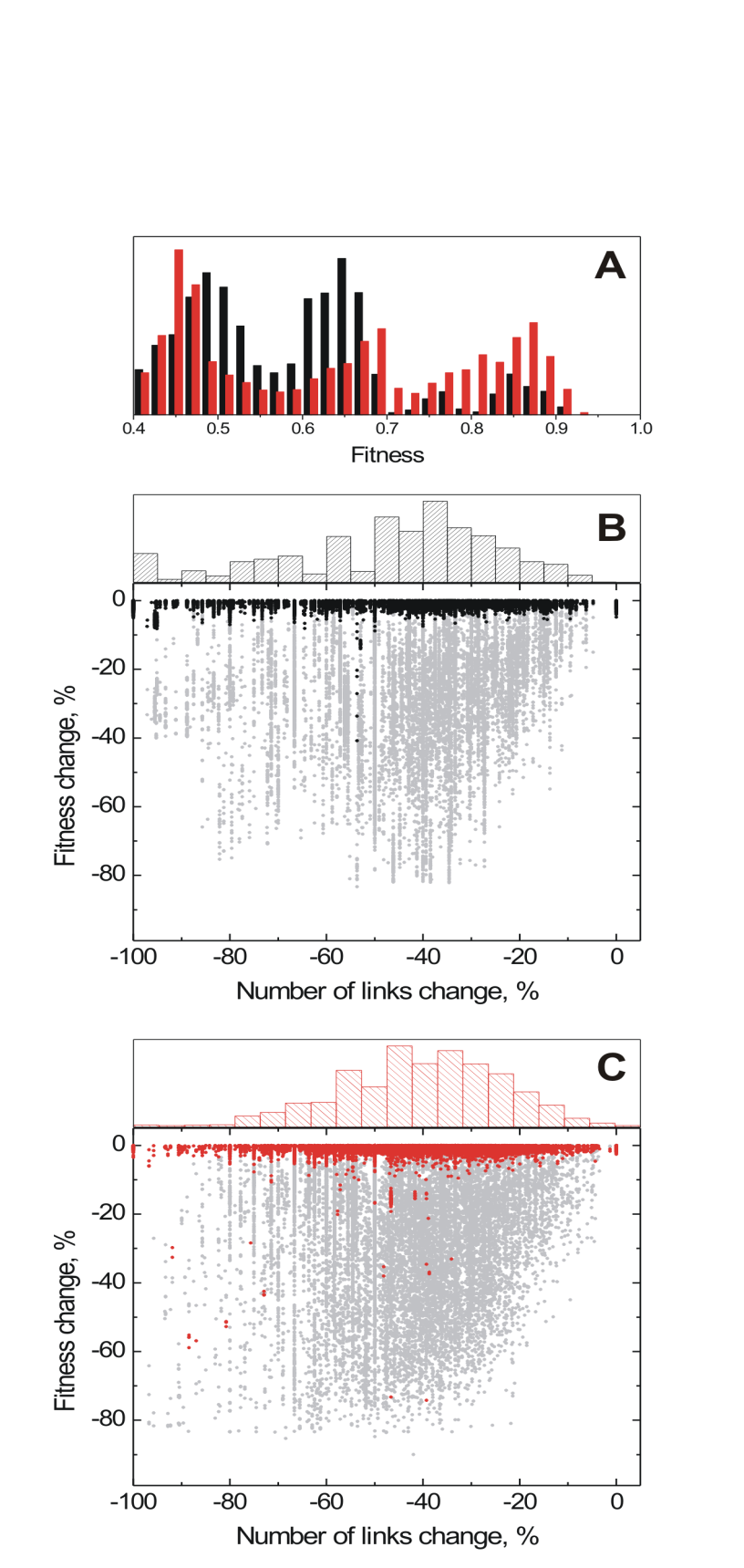


**Figure S2.** Flood-based minimization of regulatory networks of *in silico* organisms evolved in OR environments. Top panel (A) shows the distribution of fitness for cells evolved in high mutation rates (red) and low mutation rates (black). Dot plots show the statistics of the flood minimization for populations of cells evolved in OR low mutation rate (B) and OR high mutation rate (C) environments. Gray dots show the effect on fitness of a random network minimization to the same degree as obtained by the flood analysis. Bar plots in (B and C) show the distribution of minimization degree (decrease in number of links) for each type of evolved cells.


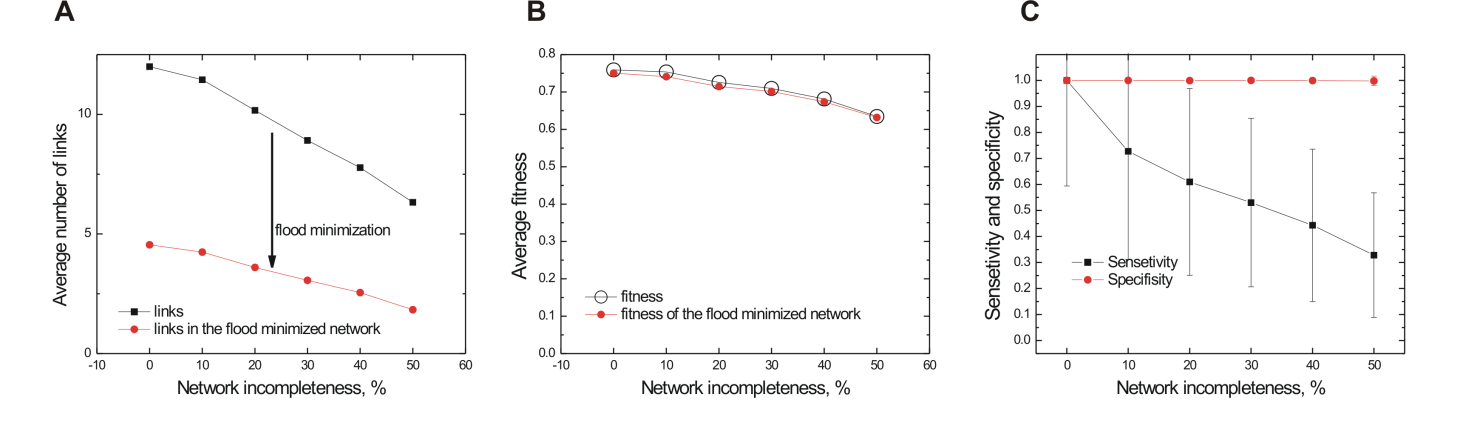


**Figure S3. Effect of the network incompleteness on the network minimization analysis for cells evolved in AND environments (**A) An average number of links in a full and flood minimized network; (B) effect of network minimization on fitness of a cell; (C) sensitivity and specificity of flood and exhaustive minimization

**Supplementary Tables**

| **Gene Ontology term** | **p-value** | **Gene Ontology Term Description** |
| --- | --- | --- |
| GO:0043234 | 2.75E-12 | protein complex |
| GO:0009060 | 3.64E-12 | aerobic respiration |
| GO:0051234 | 8.89E-12 | establishment of localization |
| GO:0006810 | 8.24E-11 | transport |
| GO:0045333 | 2.67E-09 | cellular respiration |
| GO:0009061 | 7.59E-09 | anaerobic respiration |
| GO:0055114 | 7.59E-09 | oxidation-reduction process |
| GO:0006935 | 2.28E-08 | chemotaxis |
| GO:0008137 | 3.38E-08 | NADH dehydrogenase (ubiquinone) activity |
| GO:0050136 | 3.38E-08 | NADH dehydrogenase (quinone) activity |
| GO:0042330 | 4.22E-08 | taxis |
| GO:0044424 | 4.22E-08 | intracellular part |
| GO:0009425 | 5.51E-08 | bacterial-type flagellum basal body |
| GO:0006091 | 7.91E-08 | generation of precursor metabolites and energy |
| GO:0009082 | 9.90E-08 | branched chain family amino acid biosynthetic process |
| GO:0044444 | 1.07E-07 | cytoplasmic part |
| GO:0005737 | 1.08E-07 | cytoplasm |
| GO:0048870 | 1.08E-07 | cell motility |
| GO:0008643 | 1.30E-07 | carbohydrate transport |
| GO:0048038 | 1.30E-07 | quinone binding |
| GO:0001539 | 1.77E-07 | ciliary or flagellar motility |
| GO:0044461 | 1.86E-07 | bacterial-type flagellum part |
| GO:0019861 | 2.10E-07 | flagellum |
| GO:0009288 | 2.95E-07 | bacterial-type flagellum |
| GO:0006099 | 2.95E-07 | tricarboxylic acid cycle |
| GO:0046356 | 2.95E-07 | acetyl-CoA catabolic process |
| GO:0048037 | 4.15E-07 | cofactor binding |
| GO:0015399 | 8.07E-07 | primary active transmembrane transporter activity |
| GO:0015453 | 8.07E-07 | oxidoreduction-driven active transmembrane transporter activity |
| GO:0043064 | 1.07E-06 | flagellum organization |
| GO:0044425 | 1.16E-06 | membrane part |
| GO:0043232 | 1.31E-06 | intracellular non-membrane-bounded organelle |
| GO:0016651 | 1.40E-06 | oxidoreductase activity, acting on NADH or NADPH |
| GO:0071702 | 1.54E-06 | organic substance transport |
| GO:0046914 | 1.73E-06 | transition metal ion binding |
| GO:0043623 | 2.13E-06 | cellular protein complex assembly |
| GO:0000041 | 2.26E-06 | transition metal ion transport |
| GO:0017004 | 2.40E-06 | cytochrome complex assembly |
| GO:0005506 | 2.51E-06 | iron ion binding |

**Table S1.** Gene Ontology terms and their p-value representation, along with a description of the cellular processes they participate in, for the exponential phase scenario.

| **Gene Ontology term** | **p-value** | **Gene Ontology Term Description** |
| --- | --- | --- |
| GO:0003954 | 0.00E+00 | NADH dehydrogenase activity |
| GO:0015399 | 0.00E+00 | primary active transmembrane transporter activity |
| GO:0015453 | 0.00E+00 | oxidoreduction-driven active transmembrane transporter activity |
| GO:0030964 | 0.00E+00 | NADH dehydrogenase complex |
| GO:0045271 | 0.00E+00 | respiratory chain complex I |
| GO:0045272 | 0.00E+00 | plasma membrane respiratory chain complex I |
| GO:0070470 | 0.00E+00 | plasma membrane respiratory chain |
| GO:0008137 | 1.31E-12 | NADH dehydrogenase (ubiquinone) activity |
| GO:0050136 | 1.31E-12 | NADH dehydrogenase (quinone) activity |
| GO:0043234 | 2.64E-12 | protein complex |
| GO:0016651 | 3.29E-12 | oxidoreductase activity, acting on NADH or NADPH |
| GO:0048038 | 1.43E-11 | quinone binding |
| GO:0044425 | 2.06E-08 | membrane part |
| GO:0022904 | 2.28E-08 | respiratory electron transport chain |
| GO:0009061 | 2.68E-07 | anaerobic respiration |
| GO:0045333 | 3.01E-07 | cellular respiration |
| GO:0006119 | 5.05E-07 | oxidative phosphorylation |
| GO:0042773 | 5.05E-07 | ATP synthesis coupled electron transport |
| GO:0006096 | 1.06E-06 | glycolysis |
| GO:0016491 | 2.82E-06 | oxidoreductase activity |
| GO:0006007 | 4.66E-06 | glucose catabolic process |
| GO:0009060 | 6.67E-06 | aerobic respiration |
| GO:0019740 | 2.97E-05 | nitrogen utilization |
| GO:0044464 | 3.61E-05 | cell part |
| GO:0006865 | 4.22E-05 | amino acid transport |
| GO:0055114 | 5.47E-05 | oxidation-reduction process |
| GO:0015837 | 1.33E-04 | amine transport |
| GO:0006091 | 1.60E-04 | generation of precursor metabolites and energy |
| GO:0005626 | 2.18E-04 | insoluble fraction |
| GO:0051234 | 2.18E-04 | establishment of localization |
| GO:0005624 | 2.47E-04 | membrane fraction |
| GO:0006212 | 2.51E-04 | uracil catabolic process |
| GO:0046942 | 3.44E-04 | carboxylic acid transport |
| GO:0048037 | 4.11E-04 | cofactor binding |
| GO:0044459 | 6.07E-04 | plasma membrane part |
| GO:0019860 | 8.90E-04 | uracil metabolic process |
| GO:0005829 | 9.22E-04 | cytosol |
| GO:0006208 | 9.63E-04 | pyrimidine base catabolic process |
| GO:0003954 | 0.00E+00 | NADH dehydrogenase activity |

**Table S2.** Gene Ontology terms and their p-value representation, along with a description of the cellular processes they participate in, for the stationary phase scenario.

| **Gene Ontology term** | **p-value** | **Gene Ontology Term Description** |
| --- | --- | --- |
| GO:0043234 | 2.62E-12 | protein complex |
| GO:0051234 | 2.31E-11 | establishment of localization |
| GO:0009060 | 2.31E-11 | aerobic respiration |
| GO:0006810 | 1.18E-10 | transport |
| GO:0045333 | 1.47E-09 | cellular respiration |
| GO:0009061 | 4.63E-09 | anaerobic respiration |
| GO:0044444 | 2.44E-08 | cytoplasmic part |
| GO:0055114 | 2.44E-08 | oxidation-reduction process |
| GO:0008137 | 2.44E-08 | NADH dehydrogenase (ubiquinone) activity |
| GO:0050136 | 2.44E-08 | NADH dehydrogenase (quinone) activity |
| GO:0008643 | 2.44E-08 | carbohydrate transport |
| GO:0006091 | 4.35E-08 | generation of precursor metabolites and energy |
| GO:0009082 | 9.59E-08 | branched chain family amino acid biosynthetic process |
| GO:0048038 | 1.32E-07 | quinone binding |
| GO:0044424 | 4.76E-07 | intracellular part |
| GO:0048037 | 9.62E-07 | cofactor binding |
| GO:0005737 | 9.62E-07 | cytoplasm |
| GO:0015399 | 9.62E-07 | primary active transmembrane transporter activity |
| GO:0015453 | 9.62E-07 | oxidoreduction-driven active transmembrane transporter activity |
| GO:0044425 | 9.96E-07 | membrane part |
| GO:0043064 | 1.11E-06 | flagellum organization |
| GO:0009425 | 1.15E-06 | bacterial-type flagellum basal body |
| GO:0071702 | 1.30E-06 | organic substance transport |
| GO:0016651 | 1.41E-06 | oxidoreductase activity, acting on NADH or NADPH |
| GO:0044461 | 2.09E-06 | bacterial-type flagellum part |
| GO:0043623 | 2.22E-06 | cellular protein complex assembly |
| GO:0017004 | 2.62E-06 | cytochrome complex assembly |
| GO:0006099 | 3.04E-06 | tricarboxylic acid cycle |
| GO:0046356 | 3.04E-06 | acetyl-CoA catabolic process |
| GO:0048870 | 5.31E-06 | cell motility |
| GO:0005506 | 6.91E-06 | iron ion binding |
| GO:0019861 | 9.72E-06 | flagellum |
| GO:0009432 | 9.72E-06 | SOS response |
| GO:0043232 | 1.08E-05 | intracellular non-membrane-bounded organelle |
| GO:0046914 | 1.40E-05 | transition metal ion binding |
| GO:0005829 | 1.42E-05 | cytosol |
| GO:0009288 | 1.96E-05 | bacterial-type flagellum |
| GO:0003954 | 1.96E-05 | NADH dehydrogenase activity |
| GO:0043234 | 2.62E-12 | protein complex |

**Table S3.** Gene Ontology terms and their p-value representation, along with a description of the cellular processes they participate in, for the transition phase scenario.
